# Mechanistic insights into a cold-adapted glucokinase with high thermal stability were revealed by site-directed spin labeling ESR

**DOI:** 10.2142/biophysico.bppb-v23.0005

**Published:** 2026-02-17

**Authors:** Akane Yato, Masaki Horitani

**Affiliations:** 1 The United Graduate School of Agricultural Sciences, Kagoshima University, Kagoshima 890-0065, Japan; 2 Department of Applied Biochemistry and Food Science, Faculty of Agriculture, Saga University, Saga 840-8502, Japan

**Keywords:** cold-adapted enzyme, thermal stability, site-directed spin labeling ESR, structural flexibility

## Abstract

The structural flexibility of enzymes plays an essential role in determining their catalytic efficiency and thermal stability. Cold-adapted enzymes are typically highly flexible, resulting in high catalytic activity but low stability. Glucokinase (GK) consists of the large substrates binding domain, small catalytic domain, and hinge region that undergoes conformational changes upon substrates binding. We recently reported that the psychrophilic GK from *Pseudoalteromonas* sp. AS-131 (PsGK) exhibits both high catalytic efficiency and remarkable thermal stability compared to the mesophilic GK from *Escherichia coli* (EcGK). We also found that a disulfide bond connecting the N- and C-termini in PsGK contributes to its unusual thermal stability. However, cold adaptation mechanism of cold-adapted PsGK has remained unclear. To clarify how PsGK acquires high activity, we utilized site-directed spin labeling electron spin resonance (SDSL-ESR) spectroscopy for PsGK and EcGK in the absence and presence of substrates in the wide range of temperatures. PsGK without substrates was more flexible than EcGK. Particularly, the small domain and hinge region of PsGK were highly flexible while its large domain was relatively rigid. In contrast, EcGK showed lower entire flexibility and did not exhibit domain dependent differences. When the substrates were bound, both enzymes became more rigid, but the small domain and hinge region of PsGK was still flexible whereas its large domain was considerably rigid. These results suggest that enhancing catalytic activity requires increasing flexibility only in proper sites rather than in the entire enzyme. These findings provide insight into how cold-adapted enzymes balance activity and stability.

## Significance

SDSL-ESR was demonstrated to investigate the temperature dependence of the flexibility of cold-adapted glucokinase with high thermal stability from *Pseudoalteromonas* sp. AS-131 (PsGK) in solution and to compare its flexibility with that of mesophilic glucokinase from *Escherichia coli*. Our studies revealed that the small domain containing the catalytic active sites were found and the hinge region, which is important for conformational changes when substrates bind, were highly flexible whereas the large domain, which functions as the substrate binding site, was rigid. Therefore, we concluded that the flexibility of PsGK was well-controlled to achieve high catalytic efficiency and thermal stability.

## Introduction

Enzymatic conformational changes and flexibility are closely associated with their functional properties, including catalytic activity and stability. Cold-adapted enzymes are generally known to exhibit high catalytic activity by increasing the flexibility of the entire enzyme or specific regions [1–4]. In addition, such high flexibility is often accompanied by reduction of thermal stability [3,5]. In recent years, however, exceptions have been reported that cold-adapted enzymes could maintain high catalytic activity with high thermal stability [6–9]. These dual properties have been suggested to involve the mechanism such as low activation energy or structural designs that preserve mostly rigid with increasing local flexibility in functionally important regions. Nevertheless, direct experimental evidence of the relationship between enzymatic property and conformational flexibility remains poorly understood. To fully elucidate enzyme function, it is essential to investigate flexibility under functional conditions.

Glucokinase (GK) catalyzes the reaction of glucose and adenosine triphosphate (ATP) to glucose-6-phosphate and adenosine diphosphate (ADP), which is initial enzyme of glycolysis. Previous studies have shown that GK from *Pseudoalteromonas* sp. AS-131 (PsGK), psychrophilic bacterium isolated from the Antarctic seawater was a cold-adapted enzyme exhibiting unusually high thermal stability compared to mesophilic GK from *Escherichia coli* (EcGK) [10]. The amino acid composition, molecular interactions, and surface properties in PsGK were almost identical to those in EcGK. And the biochemical and mutagenesis studies confirmed that one of high stabilization mechanisms was identified a disulfide bond connecting the N- and C-termini (Figure S1). However, the cold adaptation mechanism has not been fully understood.

**Figure 1:**
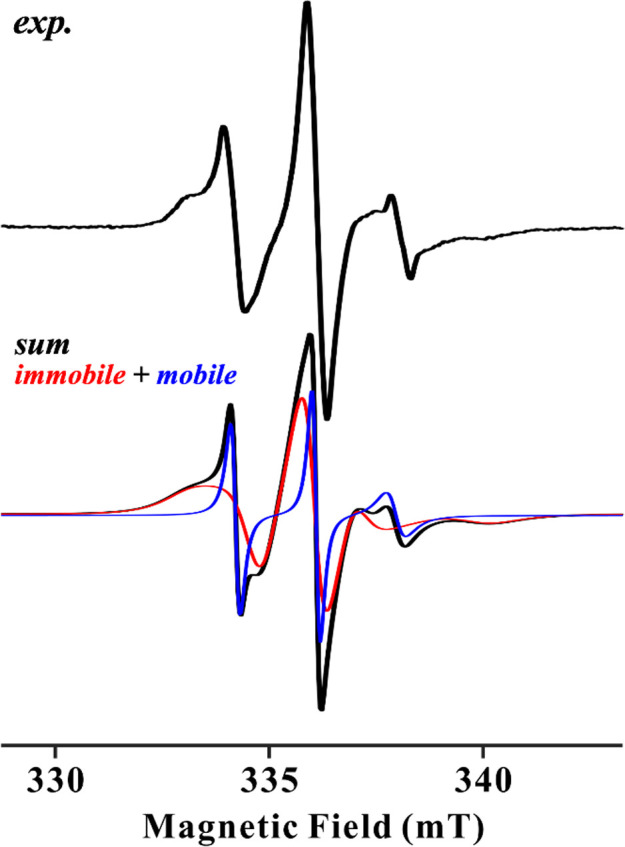
SDSL-ESR spectrum and simulation. (top) Typical SDSL-ESR spectrum was shown in black. (bottom) Simulation spectra of immobile and mobile components were shown in red and blue, respectively. Sum of simulation was shown in black.

Traditional methods, including B-factors derived from crystallography and nuclear magnetic resonance (NMR) spectroscopy, have been used to evaluate protein flexibility. However, B-factors may not adequately reflect molecular motions in solution and often require normalization procedures for comparisons between crystal structures. Likewise, NMR spectroscopy is limited by molecular size and is usually difficult to assign the signals. For a quantitative understanding of enzyme flexibility in the wide range of temperatures in solution, an approach that can directly evaluate and compare site-specific flexibility is required. Spin label reagents have stable nitric oxide radical on usually tetrapyrrole ring and can be attached with solvent accessible cysteine residues. Site-directed spin labeling electron spin resonance (SDSL-ESR) spectrum shows three hyperfine lines which is a characteristic of nitric oxide radical. SDSL-ESR spectrum of spin label incorporated into the enzymes composed of two distinct components, a mobile and an immobile component (Figure 1). The former suggests free motion of spin label, and the latter indicates a restricted environment where the spin label’s mobility is limited, reflecting on protein’s structural flexibility. These two components can be deconvoluted through spectral simulation, allowing for the estimation of their respective rotational correlation times (τ, second/rotation) and relative contributions. To evaluate local flexibility, the τ of the immobile component was discussed in this study since the τ of the mobile component did not show any temperature dependence. In addition, the τ calculated in this study was the sum of backbone and side chain of amino acid, where spin label was introduced, and these could not be decomposed. We focused on the measurements and comparison of the flexibility of secondary structure, domains and hinge region. Therefore, the large and small τ are suggested the rigid and flexible secondary structure, respectively. As far as we know, SDSL-ESR has not been used for studies on the relationship between flexibility and enzyme function and the comparison of cold-adapted enzymes with homologous enzymes.

Here, we employed SDSL-ESR to experimentally clarify site-specific differences in flexibility between PsGK and EcGK and explored how flexibility relates to the dual property of PsGK. Our results suggested that PsGK in the presence of substrates preserved flexibility in the small domain where the catalytic active site is located, and the hinge region which plays an important role in catalytic conformational change upon substrates binding while the large domain decreased flexibility where the substrates are bound. Therefore, we propose that enhancing catalytic activity requires increasing flexibility only in proper sites, such as the active site and the sites of conformational change, rather than in the entire enzyme. These findings provide new insights into how cold-adapted and thermally stable enzymes balance flexibility and stability. They also demonstrate the utility of SDSL-ESR as a powerful approach for elucidating site-specific protein flexibility in solution. This approach has promising applications in functional and engineering studies of enzymes.

## Materials and methods

### Protein preparations

Variant genes of PsGK and EcGK were designed using the wild-type PsGK and EcGK gene sequences as templates. They were synthesized by GenScript Biotech or Synbio Technologies and cloned into the expression vector pET-16b digested with *Nco*I and *Bam*HI. All proteins were expressed and purified as previously described [10]. Substrate-bound samples were prepared by adding glucose and adenylyl-imidodiphosphate (AMP-PNP) to final concentrations of 100 mM and 1 mM, respectively.

### Spin labeling

Purified PsGK and EcGK variants (20 mM Tris-HCl buffer (pH 7.6) containing 10 mM MgCl_2_ and 5 mM β-mercaptoethanol) were site-specifically labeled at their cysteine residues with the spin label (1-oxyl-2,2,5,5-tetramethyl-Δ3-pyrroline-3-methyl) methanethiosulfonate (MTSL) (Funakoshi). Purified enzymes were first applied to a desalting spin column (APRO Science Group) three times to completely remove reducing agent. In second, the enzyme was incubated with a five-molar excess of MTSL at 4°C overnight. Finally, unreacted spin labels were removed by the desalting spin column three times. The spin-labeling efficiency was determined by double integration of the ESR signal. The activity was measured for all variants using a two-step reaction as previously described [10]. The magnitudes of the activity compared to each wild type (WT) were evaluated.

### ESR spectroscopy and analysis

X-band ESR spectra were obtained using a Bruker EMX Plus spectrometer at the Institute for Molecular Science. The spectra were acquired in the CW mode at ~9.4 GHz. Temperature was controlled with a Bruker ER4121VT liquid nitrogen/gas flow cryostat system. ESR measurements were conducted in the temperature range from 5 to 50°C. We measured the activity after incubation at 50°C and confirmed that enzymes were not denatured (Table S1). Typical experimental parameters were 5 mW microwave power, 100 kHz field modulation, 5 G modulation amplitude, ~1.7 mT/s sweep rate and 10.24 ms time constant. Sample solutions (~10 μL) were loaded into capillaries (inside diameter 1.0 mm) then inserted into 4 mmφ ESR quartz tube. The immobile and mobile components were simulated using the MATLAB-based GUI SimLabel [11] to visually fit the sum of the simulations to the experimental spectrum. The τ of the immobile component calculated from simulation were analyzed using the eq. 5, as described in “Results and Discussion”.

### Calculations of the solvent-accessible surface area

The solvent-accessible surface area (SASA) was calculated using FreeSASA version 2.1.2 [12] with the Lee & Richards algorithm [13]. A probe radius of 1.4 Å and 20 slices per atom were used, corresponding to the program’s default parameters. The calculation was performed for crystal structures of the PsGK and EcGK (PDB ID: 3vpz and 1q18, respectively). Residue-wise relative solvent accessibility (RSA) values were computed based on the absolute SASA results obtained by FreeSASA, using a custom Python script (Python 3.10, pandas library).

RSA was defined as

(1) $${RSA}_{i}=\frac{{SASA}_{i}}{{SASA}_{i}^{\max}}$$

where ${SASA}_{i}^{\max}$ is the maximum solvent-accessible surface area of residue *i* in an extended tri-peptide conformation (Ala–X–Ala), according to Tien et al. [14]. The maximum SASA values for each amino acid were Ala (129.0 Å^2^), Arg (274.0 Å^2^), Asn (195.0 Å^2^), Asp (193.0 Å^2^), Cys (167.0 Å^2^), Gln (225.0 Å^2^), Glu (223.0 Å^2^), Gly (104.0 Å^2^), His (224.0 Å^2^), Ile (197.0 Å^2^), Leu (201.0 Å^2^), Lys (236.0 Å^2^), Met (224.0 Å^2^), (240.0 Å^2^), Pro (159.0 Å^2^), Ser (155.0 Å^2^), Thr (172.0 Å^2^), Trp (285.0 Å^2^), Tyr (263.0 Å^2^), and Val (174.0 Å^2^).

## Results and discussion

### Selection of spin labeling sites

**Figure 2:**
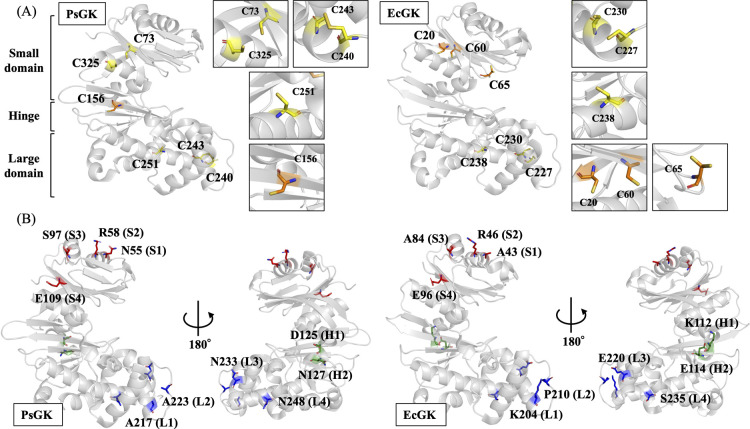
Crystal structures of PsGK (PDB ID: 3vpz) and EcGK (PDB ID: 1q18). (A) Cysteine residues were shown by sticks, and Cys156 in PsGK and Cys20, Cys60 and Cys65 in EcGK, which have been substituted with serine residues, were colored orange. The top row of insets shows cysteine residues that are retained as cysteine residues due to disulfide bond formation, the middle row shows cysteine residues that are retained as cysteine residues due to lack of solvent exposure, and the bottom row shows cysteine residues that are replaced by serine residues due to solvent exposure. (B) The positions of cysteine mutations were shown by sticks. Asn55, Arg58, Ser97 and Glu109 in PsGK and Ala43, Arg46, Ala84 and Glu96 in EcGK, Asp125 and Asn127 in PsGK, Lys112 and Glu114 in EcGK, and Ala217, Ala223, Asn233 and Asn248 in PsGK and Lys204, Pro210, Glu220 and Ser235 in EcGK are located in small domain (red sticks), hinge region (green sticks) and large domain (blue sticks), respectively. Figures prepared using PyMOL software [15].

Both PsGK wild-type (WT) and EcGK WT possess six cysteine residues and four of them are located at homologous position, Cys73, Cys240, Cys243 and Cys251 in PsGK are corresponded to Cys60, Cys227, Cys230 and Cys238 in EcGK, respectively (Figure 2A). Our previous studies revealed that Cys73 and Cys325 in PsGK were formed disulfide bond contributed to its high stability and Cys156 in PsGK and Cys20, Cys60, and Cys65 in EcGK were placed at solvent-accessible positions [10]. To prevent solvent-accessible residues from reacting with the spin-labeling reagent, MTSL, they were substituted in serine residues (Figure 2A). GK is homodimer consisting of small and large domains connected by hinge region. All spin labeled sites were selected on the surface of the homodimer enzymes and the distance between the dimeric sites was greater than 20 Å because labeled distances of less than 20 Å affect CW ESR spectrum (Table S2). Individual cysteine substitutions were introduced at four positions in the small and large domains and two positions in the hinge region in PsGK and homologous positions in EcGK as shown in Figure 2B and Table 1 and S3. To avoid confusion, we used abbreviations of each variant listed in Table 1 in this study. It was confirmed that the structural integrity of each variant was preserved by measurements of enzymatic activities (Table S1). The average labeling efficiencies were calculated to approximately 90%, estimated by UV-visual and ESR spectroscopic measurements of the protein and spin-label concentrations (Table S4).

### Temperature dependence of SDSL-ESR spectra and analysis on τ of PsGK and EcGK in the absence of substrate

**Figure 3:**
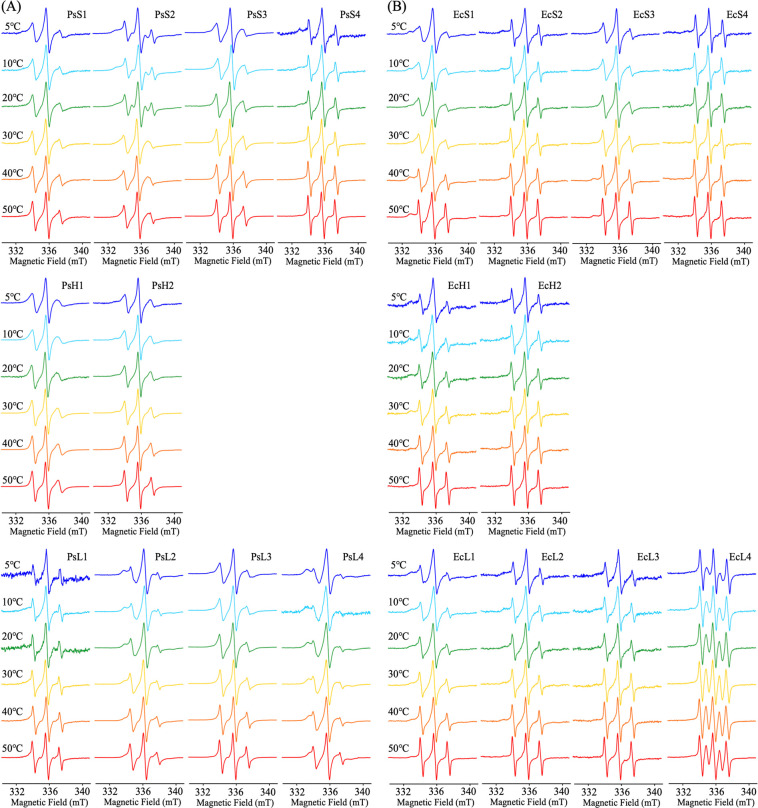
Temperature dependence of SDSL-ESR spectra for (A) PsGK and (B) EcGK labeled with MTSL. ESR spectra at 5°C, 10°C, 20°C, 30°C, 40°C, 50°C were shown in blue, light blue, green, yellow, orange and red, respectively. The conditions of ESR were described in Materials and Methods.

**Figure 4:**
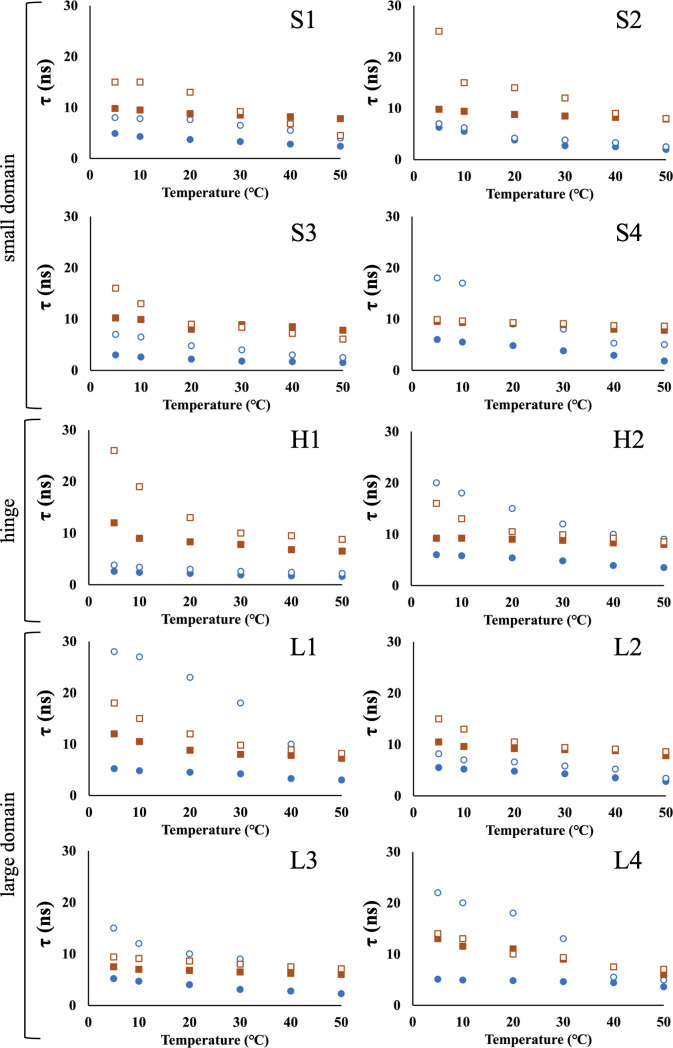
Rotational correlation times (τ) of MTSL-PsGK (blue) and MTSL-EcGK (orange) as a function of temperature in the absence and presence of substrate. The τ of PsGK and EcGK calculated from ESR spectra obtained at each temperature, were plotted as closed blue circles and closed orange squares for substrate-free conditions, and open blue circles and open orange squares for substrate-bound conditions.

Figure 3 shows the temperature dependence of SDSL-ESR spectra of PsGK and EcGK without substrate in solution. Only EcL4 showed five lines which was caused by formation of biradical as an impurity and neglectable. The corresponding calculated τ obtained at each temperature were plotted in Figure 4 (closed circles and squares). It has been reported that atomic and residual level thermal fluctuations are temperature-dependent, and that an increase in thermal energy (eq. 2) with temperature generally enhances both vibrational and structural fluctuations [16–18],

(2) $$E=k_{b}T$$

where *k*_b_ is Boltzmann constant and *T* is the absolute temperature. The τ derived from SDSL-ESR spectrum in solution was proposed to describe as a modified Stokes–Einstein–Debye equation (eq. 3) [19–23],

(3) $$\tau =\frac{\eta \left(T\right)V}{k_{b}T}k+\tau_{0}$$

where η(*T*) is the viscosity of solvent, *V* is the effective volume of the spin label, k is a dimensionless interaction parameter, and τ_0_ is the zero viscosity rotational correlation time. η(*T*) was calculated assuming the viscosity of water according to previously reported method [24]. *V* was estimated by the following equation (eq. 4),

(4) $$V=\frac{\bar{V}M}{N_{A}}$$

where $\bar{V}$ was the partial specific volume, *M* was the molecular mass of enzymes, 35.1 and 35.9 kDa for PsGK and EcGK, respectively and *N*_A_ was Avogadro constant. On the other hands, the temperature dependence of τ derived from SDSL-ESR spectrum often analyzed by the Arrhenius type relationship [25,26]. The Arrhenius type equation is given as,

(5) $$\ln\tau =\frac{U}{RT}+\ln\tau_{0}$$

where *U* is the potential energy, and *R* is the gas constant. A high potential energy suggests sensitivity to temperature, while a small τ_0_ indicates high intrinsic flexibility. In this study, the τ, *U*, and τ_0_ of PsGK and EcGK were evaluated to determine the difference in flexibility.

### The τ and Arrhrenius parameters differences between PsGK and EcGK in the absence of substrate

**Figure 5:**
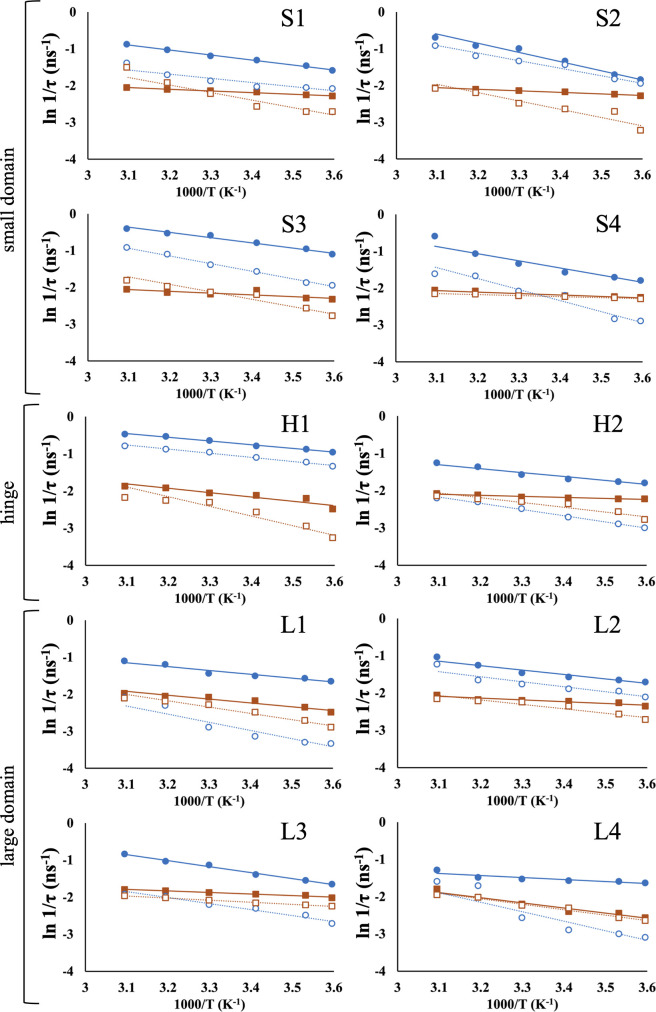
The Arrhenius plots of logarithm of 1/τ for MTSL-PsGK (blue) and MTSL-EcGK (orange) as a function of 1/temperature in the absence and presence of substrate. The substrate-free conditions represented by closed blue circles and closed orange squares, respectively, and substrate-bound conditions represented by open blue circles and open orange squares, respectively. The fitting lines were shown as solid and dashed lines for substrate-free conditions and substrate-bound conditions, respectively.

The τ of both PsGK and EcGK were decreased with temperature and PsGK consistently exhibited entirely smaller τ than EcGK (Figure 4). These observations suggested that flexibility was increased with temperature at all positions for both enzymes and PsGK entirely possessed higher flexibility than EcGK. These were consistent with the general characteristic of cold-adapted enzymes. The Arrhenius type plots were shown in Figure 5 (closed circles and squares for PsGK and EcGK, respectively) and fitting coefficient of determination were listed Table S5. To gain further insight into the property of PsGK, cold-adaptation with high thermal stability, the domain-specific analyses of structural fluctuations were conducted.

(i) *Small domain*: Calculated parameters were listed in Table 2. The small domain of PsGK showed quite small τ_0_ which were variable whereas EcGK exhibited considerably large τ_0_ which were identical. On the other hand, the potential *U* showed relatively small changes between PsGK and EcGK. These observations indicated that the flexibility in small domain of PsGK was nearly no environmental constraint and small site-specific dependent whereas the EcGK showed rigid and did not display site-specific flexibility. Flexibility is critically influenced by Intra-molecular interactions. The interaction energy in general increases in the following order, hydrogen bond<hydrophobic interaction<salt bridge. Therefore, we counted the number of those interactions in small domain which is based on the crystal structures (Table 3). Surprisingly, PsGK had a significantly larger number of hydrophobic interactions and hydrogen bonds than EcGK. Only the number of salt bridges, however, was smaller in PsGK than in EcGK. These observations suggested that salt bridges were dominant to influence flexibility or intra-molecular interactions in the crystal structure were not same as that in solutions. On the other hand, *U* showed different manners from τ_0_. The *U* of PsGK were relatively larger than those of EcGK. This suggests that the flexibility of small domain in PsGK is more sensitive to temperature. As shown in Table 4, SASA were estimated by crystal structures. Interestingly, most PsGK showed high SASA, except for PsS4. This suggests that the temperature sensitivity of flexibility might depend on the extent to which a site is exposed to the solvent. Further analysis and experiments are required to better understand the relationship between temperature sensitivity of flexibility and SASA. Additionally, a disulfide bond was formed in small domain of PsGK, contributing to high thermal stability, as previously reported (Figure S1) [10]. However, flexibility of PsGK was higher than EcGK in all range of observed temperatures. Therefore, we concluded that introducing a disulfide bond only enhances stability without loss of cold adaptation. This insight is particularly important for development of artificial enzymes. To reveal how many disulfide bonds are the best for highest thermal stability without changing or loss of activity, further research must be required.

Taken together, these results demonstrate that the small domain of PsGK contains fewer salt bridges which is an important for flexible structure. Maintaining high flexibility is important to possess high activity since the small domain contains the catalytic amino acids. A disulfide bond prevents the denaturation of high flexible small domain, resulting in high stability.

(ii) *Hinge region*: Both H1 and H2 of hinge region in PsGK showed much smaller τ_0_ values than EcGK. According to the number of intra-molecular interactions (Table 3), PsGK had a smaller number of salt bridges and hydrophobic interactions than EcGK. The difference in the number of interactions was consistent with the observation of τ_0_ for PsGK and EcGK. The small and large domains of GK convert “open” conformation to “closed” conformation upon substrates binding [27]. Therefore, the flexibility of the hinge region connecting the two domains must be important for catalytic efficiency. We propose that the highly flexible hinge region of PsGK is also important for its τ_0_ of PsH1 and PsH2, however, were larger than those of the small domain in PsGK. This may be because H1 and H2 are located on a distorted α-helix (Table S3). As shown in Table 4, the SASAs of hinge region in PsGK were much smaller than those of EcGK, suggesting that PsGK was more sensitive to temperature. However, the temperature sensitivity, *U*, for both GKs were identical or diverse, and they did not show good agreement with SASA. This suggests that the hinge region must be considered the influence from environment since the size is considerably smaller than other domains. Alternatively, the environment of hinge region may differ from the crystal structure since the entire conformation is in equilibrium between open and closed states.

(iii) *Large domain*: The τ_0_ of the large domain in PsGK was also smaller than that in EcGK. The difference between the large domain in PsGK and EcGK was smaller than the difference observed for the small domain. These results suggest that the large domain of PsGK is more flexible than that of EcGK, but more rigid than the small domain of PsGK, and its flexibility is closer to that of the large domain of EcGK. As listed in Table 3, the large domain of PsGK exhibited fewer intra-interactions than that of EcGK, particularly salt bridges and hydrophobic interactions. This observation aligns with the difference in flexibility between the two GKs. L1, L3 and L4 are located on α-helices (Table S3). On the other hand, L2 composes the outer loop of the large domain. However, τ_0_ of L2 was almost identical to other sites in the large domain of each GK. Additionally, the SASA of L2 were the smallest values in the large domain. These observations suggest that the L2 site is buried by adjacent amino acids, and that the external loop containing L2 has a rigid structure in both GKs, even though the loops are generally highly flexible. The *U* of PsGK are larger than, or almost identical to those of EcGK, except for L4. This suggests that the large domain of PsGK is more sensitive to temperature change. This means that the large domain is more likely to become a rigid structure at cold temperatures. However, the large domain of PsGK maintained higher flexibility above 5°C (Figure 4). The SASA of PsGK and EcGK were listed in Table 4. The SASA of PsL1 was smaller than EcL1, PsL2 was almost identical to EcL2, PsL3 was smaller than that of EcL3, and PsL4 was larger than that of EcL4. These tendencies did not explain the observed *U*. The SASA was estimated using the crystal structures of WT. Therefore, the SASA of the Cys variants may differ from the calculated values, and the crystallographic study of each variant is necessary to determine the accurate SASA.

Compared to τ_0_ of the small domain of PsGK, the large domain of PsGK was relatively rigid. The large domain consists of most of the enzyme and plays a role in binding substrates. Therefore, the rigid large domain contributes to high thermal stability and firm substrate binding site.

(iv) *Summary of entire structural constraints in the absence of substrate*: Our SDSL-ESR studies revealed that the structure of PsGK is entirely flexible compared to that of homologous EcGK. Interestingly, PsGK showed site-dependent intrinsic flexibility whereas EcGK exhibit less site-independent intrinsic flexibility. Especially, the small domain of PsGK was considerably highly flexible, while the large domain of PsGK was relatively rigid (Table 5). These observations are characteristic of PsGK. As we previously reported [10], PsGK exhibited cold adaptation, high catalytic efficiency, and high thermal stability. This is because (1) the catalytic amino acids were found in the flexible small domain, (2) the region that functions the conformational change on bound of substrates was the flexible hinge region, and (3) the rigid large domain was the site where most of the enzymes and the substrate binding site. On the other hand, the intrinsic flexibility of EcGK was almost identical to all parts of enzyme. Thus, we concluded that PsGK was well-designed evolutionally. This characteristic is likely important for survival in cold environments.

### Temperature dependence of SDSL-ESR spectra and analysis on τ of PsGK and EcGK in the presence of substrates

**Figure 6:**
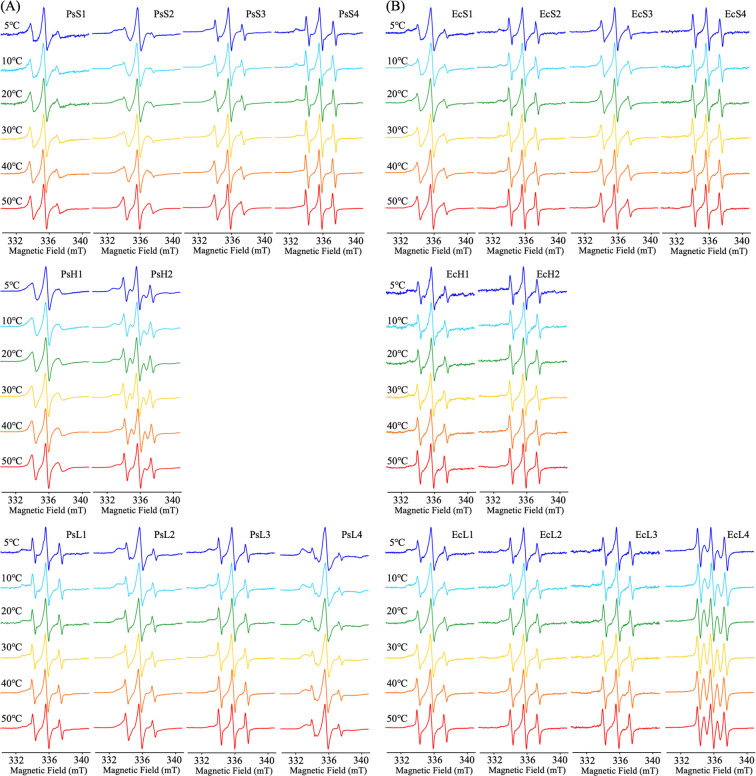
Temperature dependence of SDSL-ESR spectra for (A) PsGK and (B) EcGK labeled with MTSL in the presence of substrates. ESR spectra at 5°C, 10°C, 20°C, 30°C, 40°C, 50°C were shown in blue, light blue, green, yellow, orange and red, respectively. The conditions of ESR were described in Materials and Methods.

SDSL-ESR spectra of PsGK and EcGK with glucose and AMP-PNP which was ATP analogue in solution, recorded over the temperature range of 5–50°C, were shown in Figure 6. Like EcL4 without substrate, PsH2 and EcL4 exhibited five lines which were caused by the formation of biradical impurities that were neglectable. The immobile and mobile components of each ESR spectrum were deconvoluted by the computational simulation. The calculated τ of the immobile component was plotted with temperatures as well as substrate-unbound studies (Figure 4, open circles and squares). The τ of most position in PsGK and EcGK increased significantly with substrates binding. These observations are quite common and have been confirmed by other structural biology techniques, such as crystallography. Thus, we propose that SDSL-ESR is also a powerful tool to investigate protein flexibility.

### The τ and Arrhrenius parameters differences between PsGK and EcGK in the presence of substrates

As mentioned above, the Arrhenius type fitting for both GKs in the presence of substrates were also calculated (Figure 5, dashed lines, Table 6). To gain further insight into the properties of PsGK, which exhibits cold-adaptation with high thermal stability, the domain-specific analyses of structural fluctuations were conducted, as well as substrate-free studies.

(i) *Small domain*: The temperature sensitivity of flexibility, *U*, of the small domain in PsGK did not affect by substrates binding. This suggests that all sites do not alter their environment. The intrinsic flexibility, τ_0_, of PsS1 and PsS2 increased with substrates, while those of PsS3 and PsS4 decreased. However, PsS3 and PsS4 also became less flexible within the observed temperature range (Figure 4). The decrease in flexibility of the small domain in PsGK upon substrates binding is not surprising because enzymes generally become more compact and less flexible when substrate is bound. As discussed in the results of the SDSL-ESR studies on GK in the absence of substrate, the high flexibility of the small domain, where the catalytic amino acids are found, is required for high catalytic efficiency. Except for PsS4, the flexibility of the small domain retained a highly flexible structure in the range of observed temperatures (Figure 4). PsS4 is located on a β-sheet that connects via hydrogen bonds with other β-sheets forming a disulfide bond connecting N- and C-termini of PsGK (Figure S1 and S2). In fact, neither *U* nor τ_0_ of EcS4 changed upon substrates binding since the small domain of EcGK has no disulfide bond. Therefore, we concluded that the reduction in PsS4 flexibility is due to the adjacent tight disulfide bond, and that the small domain of PsGK retains enough flexibility for catalytic activity at cold temperatures. Interestingly, τ_0_ of EcS1, EcS2, and EcS3 decreased upon substrates binding, while temperature sensitivity, *U*, increased. This suggests that the small domain of EcGK becomes more flexible at high temperatures and more rigid at cold temperatures. This is consistent with the EcGK’s activity to exhibit high activity at high temperatures and low activity at cold temperatures. Importantly, the temperature dependence of flexibility of EcGK are identical to the temperature dependence of mesophilic EcGK activity.

(ii) *Hinge region*: The hinge region of both PsGK and EcGK in presence of substrates became rigid at all range of observed temperatures. The flexibility of PsH1 did not affect upon substrates binding in the observed temperatures whereas that of PsH2 decreased (Figure 4). Inversely, the flexibility of EcH1 dramatically increased while that of EcH2 became somewhat increasing. In another word, the structural change in PsH2 and EcH1 were induced and PsH1 and EcH2 have no changes in their environment upon substrates binding. These observations suggest that the bending position differ in each GKs when the conformation change from open to closed state by bound of substrates. In fact, comparisons of amino acids sequence revealed that the hinge region of PsGK showed only 29% homology with EcGK although the entire sequence had 35% similarities. Enzymes constantly suffer the substitution of amino acids without changing their functions in evolution. Therefore, catalytically important amino acids must be conserved, but the mutation of the hinge region would be more acceptable because the function of the hinge is more substitutable by various amino acids compared to catalytic reaction.

(iii) *Large domain*: As shown in Figure 4, the flexibility of the large domain decreased in both PsGK and EcGK by bound of substrates. Interestingly, the τ of PsGK increased considerably except at the L2 position. This suggests that the PsL1, PsL3 and PsL4 became rigid structures, while the environment of PsL2 remained no change upon substrates binding. This is a reasonable observation because only L2 is composed of a loop whereas the rest are located on α-helix (Table S3). The small domain and the hinge region of PsGK lost flexibility when substrates are bound, but most positions still maintained higher flexibility than EcGK. Conversely, the large domain of PsGK dramatically reduced flexibility, becoming more rigid than EcGK. In fact, large domain of PsGK exhibited a larger *U* than EcGK upon substrate binding, suggesting that it is more sensitive to temperature change, which means the large domain is more likely to become a rigid structure at cold temperatures. The substrates, glucose and ATP, are bound in the large domain. Thus, the rigid large domain takes advantage of efficient catalysis to accurate substrate binding since it provides the substrates binding site. We propose that the rigid large domain plays an important role in the formation of ternary complex, catalytic amino acids in small domain-glucose-ATP. Therefore, the rigid large domain of PsGK contributes to higher catalytic activity than EcGK when the substrates were bound.

(iv) *Summary of entire intrinsic constraints in the presence of substrates*: SDSL-ESR studies were also performed on PsGK and EcGK with the substrates, glucose and AMP-PNP. All positions became rigid upon substrates binding. This observation is quite common, as the entire enzyme structure changes to a more compact form by bound of substrate. Notably, the small domain and hinge region of PsGK were more flexible than those of EcGK. Meanwhile, the large domain of PsGK was more rigid than that of EcGK. The small domain contains catalytically important residues, and the hinge region plays an important role in the conformational change from open to closed states when substrates are bound. On the other hand, the large domain provides rigid substrate binding sites. Therefore, the high flexibility of the small domain and hinge region and the rigid large domain are important for high catalytic efficiency of PsGK.

## Conclusion

Up to date, the enzymes from thermophile are developed for industrial and research use because of their high stability. However, cold-adapted enzymes which have much higher catalytic efficiency than thermostable enzymes have been rarely used. This is because cold-adapted enzymes are considered to have low stability. Recently we have reported cold-adapted enzymes with high stability [10]. To figure these mechanisms out provide insights into development of high active enzyme with high stability. Studying enzyme flexibility is important to understand the relationship between the activity and stability since structural flexibility involves activity and stability.

In this study, temperature dependent of SDSL-ESR measurements were performed on the cold-adapted and highly thermally stable enzyme, PsGK, and the mesophilic homologous enzyme, EcGK, as comparison. In the absence of substrate, PsGK exhibited entirely higher flexibility than EcGK. Interestingly, PsGK showed domain specific flexibility, particularly the small domain and hinge regions were highly flexible and the large domain was relatively rigid. Conversely, the flexibility of EcGK did not show domain specific dependence. Altogether of our observations, PsGK maintains low-temperature reactivity by preserving flexibility in the small domain and hinge regions and high thermal stability with rigid the large domain. Thus, PsGK appears to employ a unique dynamic design principle that reconciles both low-temperature activity and high-temperature stability. This study provides important insights into the cold adaptation mechanisms and the molecular design of high active enzyme with high stability. We also performed SDSL-ESR studies on PsGK and EcGK in the presence of substrates. Our results confirmed that both enzymes became rigid structures when substrates were bound. Surprisingly, the flexibility of the large domain decreased dramatically in PsGK, while the small domain and hinge region remained flexible. We proposed that all these changes in PsGK were important to enhance the activity. In contrast, the flexibility of both domains and hinge region of EcGK changed ordinarily upon substrates binding. Therefore, the observed flexibilities derived from SDSL-ESR were a good match for the temperature dependence of the activity.

In this study, we demonstrated that SDSL-ESR is a powerful biophysical technique for investigating temperature dependent enzyme flexibility. The analysis of temperature dependent flexibility provides valuable insights into temperature adaptation mechanisms and thermal stability. In case of thermally stable cold-adapted PsGK, the balance of flexibility and rigidity were adjusted to achieve high catalytic efficiency and high stability.

## Conflict of interest

The authors declare no conflicts of interest.

## Author contributions

AY conducted all experiments. MH conceived this work. AY and MH analyzed and discussed the results and wrote the manuscript.

## Data availability

The evidence data generated and/or analyzed during the current study are available from the corresponding author on reasonable request.

## Figures and Tables

**Table 1:** Mutational positions and abbreviations in PsGK and homologous positions in EcGK

	PsGK		EcGK	
	amino acid number	abbreviation	amino acid number	abbreviation
small domain	N55	PsS1	A43	EcS1
	R58	PsS2	R46	EcS2
	S97	PsS3	A84	EcS3
	E109	PsS4	E96	EcS4
hinge region	D125	PsH1	K112	EcH1
	N127	PsH2	E114	EcH2
large domain	A217	PsL1	K204	EcL1
	A223	PsL2	P210	EcL2
	N233	PsL3	E220	EcL3
	N248	PsL4	S235	EcL4

**Table 2:** Fitting parameters for PsGK and EcGK in the absence of substrate

	*U* (kJ/mol)	τ_0_ (ns)		*U* (kJ/mol)	τ_0_ (ns)
PsS1	11±0.51	0.036±0.0075	EcS1	3.7±0.22	1.9±0.17
PsS2	21±1.4	0.00081±0.00048	EcS2	3.5±0.24	2.1±0.20
PsS3	12±0.88	0.018±0.0064	EcS3	3.4±1.3	1.8±0.97
PsS4	16±2.1	0.0059±0.0051	EcS4	3.3±0.51	2.3±0.49
PsH1	8.3±0.36	0.071±0.011	EcH1	9.7±2.0	0.16±0.14
PsH2	8.7±1.0	0.14±0.060	EcH2	2.3±0.34	3.4±0.47
PsL1	8.6±1.1	0.13±0.057	EcL1	8.6±1.2	0.27±0.13
PsL2	9.8±1.3	0.080±0.042	EcL2	4.0±0.68	1.8±0.51
PsL3	13±0.56	0.016±0.0037	EcL3	3.5±0.32	1.6±0.21
PsL4	4.5±1.0	0.074±0.32	EcL4	11±1.2	0.093±0.047

**Table 3:** The number of intra-molecular interactions of each domain in PsGK and EcGK

	PsGK			EcGK		
	small	hinge	large	small	hinge	large
Salt bridge	3	0	14	5	1	51
Hydrophobic interaction	140	24	164	100	28	181
Hydrogen bond	70	10	101	64	9	103

**Table 4:** The solvent-accessible surface areas (SASA) of each mutated site in PsGK and EcGK

PsGK (Å^2^)		EcGK (Å^2^)	
PsS1	69.61	EcS1	26.28
PsS2	130.42	EcS2	120.27
PsS3	83.41	EcS3	63.03
PsS4	32.29	EcS4	49.57
PsH1	45.62	EcH1	139.14
PsH2	74.91	EcH2	125.08
PsL1	48.80	EcL1	76.85
PsL2	38.01	EcL2	39.53
PsL3	88.47	EcL3	115.04
PsL4	59.04	EcL4	40.71

**Table 5:** Summary of regional flexibility

region	Substrate	flexibility*	
		PsGK	EcGK
small domain	without	+++ ~ +++++	+
	with	++ ~ +++++	+ ~ +++
hinge	without	++ ~ +++	+ ~ ++
	with	++ ~ +++	++~ ++++
large domain	without	++ ~ +++	+ ~ ++
	with	+++ ~ ++++	+ ~ +++

*The more “+” indicated, the greater the flexibility.

**Table 6:** Fitting parameters for PsGK and EcGK in the presence of substrates

	*U* (kJ/mol)	τ_0_ (ns)		*U* (kJ/mol)	τ_0_ (ns)
PsS1	9.4±1.9	0.15±0.12	EcS1	17±2.8	0.010±0.012
PsS2	17±1.5	0.0043±0.0027	EcS2	19±4.2	0.0067±0.012
PsS3	17±0.79	0.0041±0.0014	EcS3	17±2.0	0.011±0.0090
PsS4	25±3.1	0.00040±0.00053	EcS4	2.3±0.16	3.6±0.23
PsH1	9.2±0.52	0.069±0.015	EcH1	22±3.3	0.0021±0.0030
PsH2	8.7±0.39	0.14±0.0082	EcH2	11±1.8	0.15±0.11
PsL1	18±3.1	0.011±0.015	EcL1	14±1.3	0.039±0.021
PsL2	11±1.8	0.065±0.049	EcL2	9.9±1.5	0.20±0.12
PsL3	14±1.4	0.042±0.025	EcL3	4.7±0.12	1.2±0.062
PsL4	21±4.4	0.0025±0.0045	EcL4	12±0.87	0.071±0.026
