# Supplementary material for: Mechanistic insights into a cold-adapted glucokinase with high thermal stability were revealed by site-directed spin labeling ESR
Source: Biophys Physicobiol. 2026 Feb 17;23(1):e230005. doi: 10.2142/biophysico.bppb-v23.0005 (PMC13077128; doi:10.2142/biophysico.bppb-v23.0005)
Supplement: Supplementary file 1 — Supplementary Materials [file 23_e230005_1.pdf]

Table S1. Relative activities of 20 and 50°C compared to WT

	Relative activities (%)	
	PsGK	EcGK
WT	100	100
S1	50.6	63.5
S2	115	45.9
S3	142	144
S4	202	48.9
H1	208	92.7
H2	191	80.5
L1	177	103
L2	111	38.5
L3	52.8	107
L4	65.5	128

Table S2. Distances between spin labels in dimers of PsGK and EcGK

	Distances between spin labels (Å)	
	PsGK	EcGK
S1	79.1	85.8
S2	72.6	81.4
S3	42.2	52.0
S4	23.1	32.8
H1	58.4	50.3
H2	65.3	66.0
L1	32.8	29.6
L2	43.4	47.4
L3	71.3	69.9
L4	45.2	39.2

Table S3. The location of labels

	location	
	PsGK	EcGK
S1	α-helix	α-helix
S2	α-helix	α-helix
S3	α-helix	α-helix
S4	β-sheet	β-sheet
H1	distorted α-helix	distorted α-helix
H2	distorted α-helix	distorted α-helix
L1	α-helix	α-helix
L2	loop	loop
L3	α-helix	α-helix
L4	α-helix	α-helix

Table S4. Labeling efficiencies of PsGK-MTSL and EcGK-MTSL

	Labeling efficiencies (%)	
	PsGK	EcGK
S1	94	131
S2	56	46
S3	158	92
S4	100	37
H1	73	44
H2	124	59
L1	97	83
L2	76	49
L3	104	40
L4	41	42

Table S5. The fitting coefficient of determination of PsGK and EcGK for substrate (S)-free and S-bound condition

	**Fitting coefficient of determination**			
	PsGK		EcGK	
	S-free	S-bound	S-free	S-bound
S1	0.9928	0.8697	0.9868	0.9235
S2	0.9860	0.9743	0.9823	0.8548
S3	0.9800	0.9932	0.6890	0.9511
S4	0.9498	0.9582	0.9132	0.9825
H1	0.9928	0.9882	0.8541	0.9277
H2	0.9533	0.9972	0.9238	0.9006
L1	0.9464	0.9205	0.9307	0.9689
L2	0.9447	0.9139	0.8966	0.9219
L3	0.9940	0.9601	0.9677	0.9973
L4	0.8288	0.8958	0.9607	0.9819

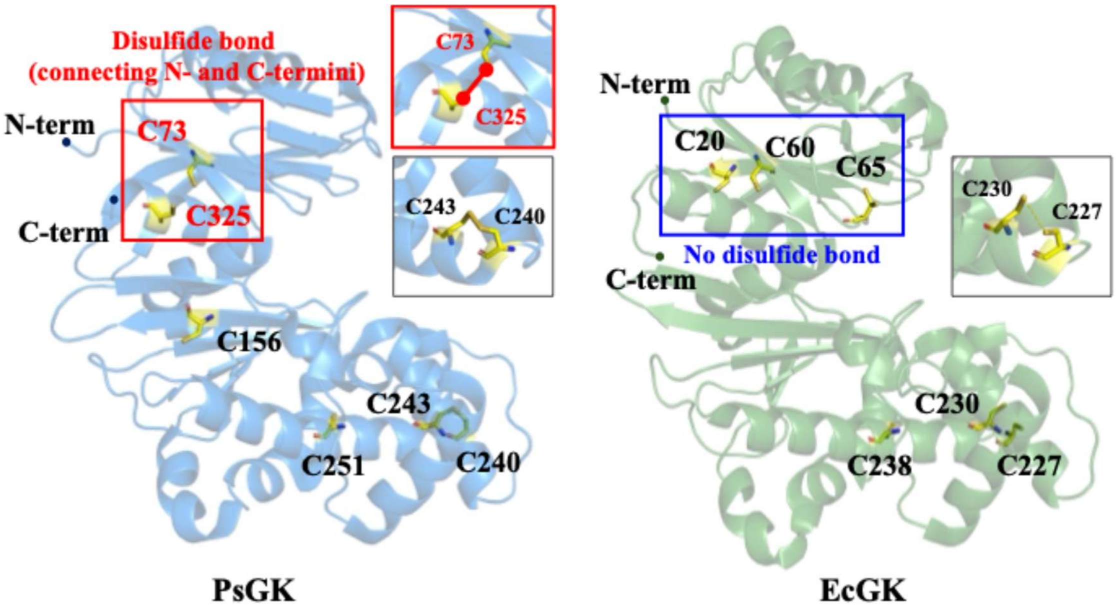

Figure S1. The positions of cysteine residues in the crystal structure of PsGK and EcGK.
Cys73 and Cys325 in PsGK forms the disulfide bond connecting N- and C-termini in solution, which contributes its high thermal stability.

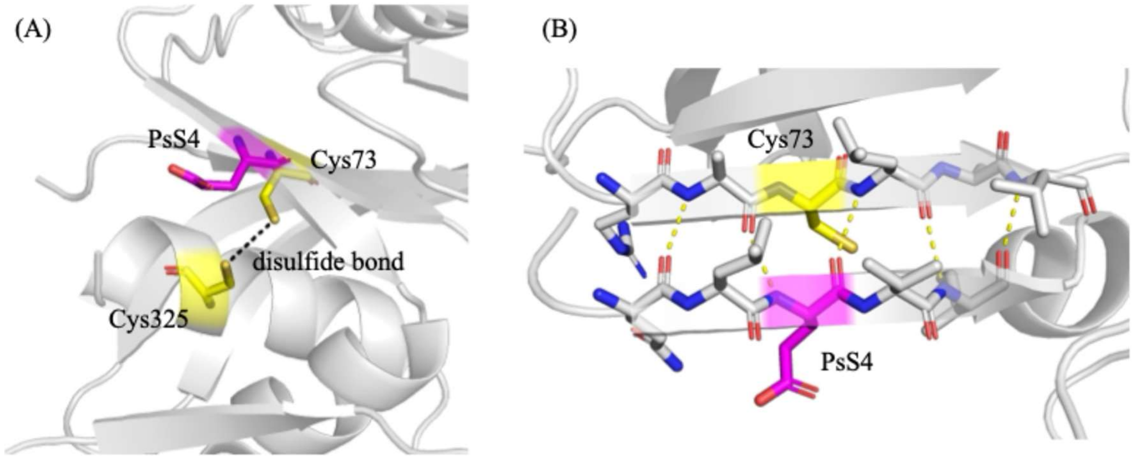

Figure S2. Structure of PsGK around the β-sheet where PsS4 is located. (A) Disulfide bond around PsS4. PsS4 is shown in magenta, and the cysteine residues which forms a disulfide bond are shown in yellow. (B) The β-sheets containing PsS4 and Cys73. These β-sheets interact with each other through hydrogen bonds (yellow dashed line).
